# Anticipatory nausea in cyclical vomiting

**DOI:** 10.1186/1471-2431-5-3

**Published:** 2005-03-24

**Authors:** Fiona E McRonald, David R Fleisher

**Affiliations:** 1 Department of Clinical Dental Sciences, University of Liverpool, Daulby Street, Liverpool, L69 3GN, UK; 2 Department of Child Health, University of Missouri School of Medicine, One Hospital Drive Columbia MO 65212, USA

## Abstract

**Background:**

Cyclical Vomiting Syndrome (CVS) is characterised by discrete, unexplained episodes of intense nausea and vomiting, and mainly affects children and adolescents. Comprehending Cyclical Vomiting Syndrome requires awareness of the severity of nausea experienced by patients. As a subjective symptom, nausea is easily overlooked, yet is the most distressing symptom for patients and causes many behavioural changes during attacks.

**Case presentation:**

This first-hand account of one patient's experience of Cyclical Vomiting Syndrome shows how severe nausea contributed to the development of anticipatory nausea and vomiting (ANV), a conditioned response frequently observed in chemotherapy patients. This conditioning apparently worsened the course of the patient's disease. Anticipatory nausea and vomiting has not previously been recognised in Cyclical Vomiting Syndrome, however predictors of its occurrence in oncology patients indicate that it could complicate many cases.

**Conclusion:**

We suggest a model whereby untreated severe and prolonged nausea provokes anxiety about further cyclical vomiting attacks. This anxiety facilitates conditioning, thus increasing the range of triggers in a self-perpetuating manner. Effective management of the nausea-anxiety feedback loop can reduce the likelihood of anticipatory nausea and vomiting developing in other patients.

## Background

Cyclical Vomiting Syndrome (CVS) has been recognised for over 100 years [1]. It is probably a manifestation of migraine diathesis [2], and is characterised by recurrent episodes of intense [3,4] nausea and vomiting, with normal health between emetic crises. Attacks are self-limiting, lasting several hours to over a week, and their duration is often stereotypical for each patient [5]. Episodes of CVS are most commonly precipitated by infections, physical or emotional stress, excitement, or specific foods [6], and often commence during the night or early morning [3,6].

The following case report is that of the first author. It is derived from patient memory and family diaries, and describes a previously unrecognised complication of CVS that is invisible to the clinician due to its subjectivity. It suggests a triggering mechanism based upon classical conditioning and exacerbated by anxiety about the prospect of severe nausea, similar to the anticipatory nausea and vomiting (ANV) seen in chemotherapy patients. Recognition and effective management of nausea can reduce the risk of this complication from developing in other CVS patients.

## Case report

A 28-year-old postdoctoral researcher experienced multiple episodes of vomiting from the age of 3 years. The episodes occurred with upper respiratory tract infections and asthma, and generally lasted 24–48 hours. The patient's mother had nearly identical episodes during childhood. The maternal grandmother experienced hyperemesis gravidarum. The patient's sister and paternal grandfather had classical migraine headaches.

The patient first presented to the hospital at age 10 1/2 years. A typical vomiting attack had been triggered by a cold, but had not resolved after four days. Despite treatment with intravenous fluids, the vomiting continued for a further two days before resolving. Cyclical vomiting was diagnosed. She was subsequently hospitalised repeatedly with episodes of about four days duration, with vomiting occurring every 5 to 10 minutes at peak. She continued to have similar attacks during every infection, but *never *without prior infection. The symptoms were refractory to anti-emetics available at the time (eg. metoclopramide, prochlorperazine).

Shortly before Christmas of that year (1986), a 6 1/2-day vomiting episode was triggered by pneumonia, and the patient spent the Christmas period in hospital. The following year (1987), a cold triggered a CVS attack two days before Christmas. Having spent two successive Christmases in hospital, the patient hoped to be well the next year. However, she was woken by nausea in the early hours of Christmas Day 1988 and began vomiting. Uniquely, this episode had not been triggered by an infection, and the patient (then aged thirteen) recognised this. Subsequently, episodes rapidly increased in frequency: compared with an average of 7.3 episodes per year in the three preceding years, the patient averaged 22.3 episodes per year over the three years following Christmas 1988. Many further episodes now occurred without infection, but coincided with visits to certain places (e.g. friend's house), or commenced on specific days of the week (e.g. Saturday). Most episodes resulted in 3–8 day hospitalisations. At one point, a repetitive cycle commenced, where each episode occurred exactly two days after discharge from hospital following the previous attack. The patient was aware of this pattern and consciously afraid of becoming ill at the expected time. Her attacks always commenced nocturnally and she became reluctant to go to sleep, fearful of another episode beginning.

The patient experienced exhaustion and severe, intractable nausea for the entire length of her emetic episodes (up to 6 1/2 days), and the nausea caused marked behavioural changes. Sleep was the only state that allowed her to be insensible to nausea, but she was often unable to lie down, as this intensified her nausea, causing retching and heartburn. Instead, she spent hours sitting motionless in bed with her hips and knees flexed, her arms and head resting on her knees and her eyes closed. She was unable to lie supine for abdominal palpation. She would repeatedly expectorate, as she experienced hypersalivation [7] and was too nauseated to swallow. Accumulated oral secretions, and the profound fatigue and confusion that accompany nausea, contributed to her verbal unresponsiveness. Attempting to reduce intolerable nausea, she frequently induced vomiting by drinking large volumes of water. This also diluted bile and gastric acid, thus lessening oesophageal and oropharyngeal discomfort during vomiting [7].

Consistent with the natural history of the condition, the attacks gradually became less frequent as the patient grew older. Availability of newer anti-emetic agents (5-HT_3 _antagonists) also contributed to the improvement: prompt treatment of episodes with intravenous ondansetron, ranitidine and analgesics reduced the duration and severity of attacks, and the same medications were partially successful as prophylactics. Thus, having achieved partial symptom control, and an understanding of how her anticipation of episodes was exacerbating her condition (and therefore when to use prophylactic medication), the patient felt more in control of her illness, feared it less, and conditioned responses to situations previously associated with CVS attacks gradually diminished. To date, the patient has been hospitalised nearly 100 times, however now experiences vomiting attacks only rarely (fifteen episodes over the past decade, including only one episode over the past five years).

## Discussion

The emetic reflex has evolved to expel ingested toxins [4]. Nausea is a vital component of this protective reflex: as an intensely unpleasant sensation it subsequently causes potent aversion to the offending food through association. Thus a key feature of nausea is its rapid conditioning, and non-vomiting species (e.g. rodents) rely solely upon this 'conditioned taste-aversion' for avoiding toxins. Unfortunately, this evolutionary legacy is problematic in clinical situations. Oncology patients whose vomiting is caused by cytotoxic drugs may also develop emesis *prior *to subsequent treatments [8]. This anticipatory nausea and vomiting (ANV) occurs through classical (Pavlovian) conditioning [8,9]. Chemotherapy (the unconditioned stimulus) is administered in hospital (the conditioned stimulus). Chemotherapy causes emesis (the unconditioned response). Patients subsequently associate the hospital with nausea and vomiting: smells, sights, or thoughts of the hospital can then elicit emesis (the conditioned response) without the emetogenic agent.

ANV is also seen in animal models [10], and is possibly involved in pregnancy-sickness [11]. However, its occurrence in CVS has remained unrecognised. In this patient's case, ANV increasingly precipitated CVS episodes. Before Christmas 1988, attacks *only *occurred during infections. Christmas (associated with infection and vomiting in two successive years) was the first conditioned stimulus. Christmas subsequently elicited the conditioned response (vomiting) in the absence of the unconditioned stimulus (infection). Occurrence of attacks without prior infection caused the patient to fear the illness and search for alternative triggers. A concurrent increase in the frequency of episodes occurred, and increasingly insignificant events associated with past episodes became sufficient to trigger vomiting. This 'stimulus generalisation', whereby conditioned stimuli become progressively less specific, is a feature of conditioned responses [9]: in some oncology patients the sight of *any *nurse can eventually induce emesis [9]. In our CVS patient, fear or expectation of an attack *in itself *became a trigger, and she became conscious of this. Consequently, many attacks occurred before important occasions (e.g. holidays, family celebrations, school examinations, university interviews), when she particularly wished to remain well: this constantly reinforced conditioning. As her episodes always commenced during sleep, she felt powerless to control her ANV.

One study [12] found ANV in 59% of paediatric cancer patients. Development of ANV is positively correlated with severity of vomiting (intensity, frequency, duration) and number of chemotherapy cycles ('conditioning trials'); and inversely with patient age [9,13]. In CVS, the severity of emesis [4] and number of conditioning trials can exceed that in chemotherapy patients, and CVS mainly affects children. Based on these predictors, and the intrinsic evolutionary links between conditioning and nausea, other CVS patients might develop ANV, as illustrated by another young woman's description: " [For] about two months...I would get sick every Saturday morning. I would be sick until Wednesday, feel good on Thursday and Friday and then the cycle would start all over. Looking back at it now, I know I worried when next Saturday would roll around. I think I worried myself so much that in a way I helped my body into the cycle." [14]. We believe that ANV could complicate many CVS cases in addition to these two, but is extremely difficult for clinicians to recognise. Whilst triggers for chemotherapy-associated ANV are specific, controlled, and can be objectively observed by doctors within the hospital; the conditioned stimuli for CVS-associated ANV are subtle (e.g. certain day of the week), subjective (e.g. fear of a CVS attack), and occur outside of the hospital, thus are invisible to doctors. As a first-hand account, this case report therefore provides a unique, qualitative perspective on triggering factors as viewed through the eye of the patient, and reveals the hidden role of conditioning.

ANV progressed in this patient because of her extremely severe nausea, and her consequent fear of future episodes. Conditioning is more potent when the patient is anxious [13,15,16]; and in patients who expect and experience much distress from nausea [12]. Based on these observations, therapy to prevent ANV in CVS should have two aims: reduction of nausea, and reduction of anxiety. Consistent with this, it is noted that CVS patients who are given prompt anti-nausea treatment during an attack experience recurrence of attacks less frequently [7].

Unfortunately, most CVS patients are treated by non-specialists, who may overlook nausea as it is subjective and unquantifiable [17,18]. Standard management therefore focuses on rehydration, which, without controlling nausea, cannot prevent dread of future attacks and the development of ANV. Iatrogenic anxiety may exacerbate this: nausea-induced behavioural changes seen during attacks [2,7], which may appear 'psychotic' [19] and 'regressive' [20], can prompt the misdiagnosis of factitious vomiting [2], hence raising patient anxiety through stigmatisation. For example, the patient reported here feared being ill before school examinations not only because of the physical unpleasantness of the illness and the educational consequences of missing important exams, but also because she believed that her doctors would interpret a CVS attack at such a time as evidence of psychosomatic illness.

Management of nausea should comprise an individualised plan devised between patient and physician. Where attacks are sufficiently frequent to justify daily medication, anti-migraine prophylaxis (e.g. cyproheptadine, pizotifen, amitriptyline or propranolol) might help prevent episodes. Prophylaxis also requires amelioration of known triggering factors, e.g. acute or chronic infection. In patients with prodromal symptoms, oral ondansetron and/or lorazepam sometimes abort the episode. If vomiting commences, an intravenous infusion containing glucose, sodium, potassium and ranitidine should be started immediately. Intravenous ondansetron and lorazepam may terminate vomiting; otherwise the patient should be sedated to reduce sensations of nausea. This can be achieved using intravenous chlorpromazine plus diphenhydramine every 3 to 4 hours until the episode abates.

The second goal of therapy, reduction of anxiety about the illness, will result from successful treatment of nausea, but is also facilitated by a patient-centred, holistic approach to care. Established ANV can be treated by relaxation-based behavioural approaches such as 'counterconditioning' (systematic desensitisation) and hypnosis [11]. However, as ANV is mediated through the normal psychological process of classical conditioning, it should be regarded as a normal response to severe nausea, and not as a primary anxiety disorder (although pre-existing anxiety could exacerbate ANV). Oncology patients often assume that ANV is indicative of something psychologically wrong with them (which causes further anxiety), and are therefore reluctant to report it to hospital staff [21], so reassurance of its normality is important.

## Conclusion

This report illustrates the potential importance of ANV as a self-perpetuating triggering mechanism in CVS, and describes the positive feedback loop between nausea and anxiety. Conditioning might contribute to regular timing of episodes in some patients [2], account for seemingly implausible triggers, and explain attacks that superficially appear to be triggered by excitement or stress (e.g. Christmas, exams). Its prevalence in other CVS patients should therefore be investigated. Correct management of CVS should help to prevent ANV, and to reverse it where it has already arisen.

## Competing interests

The author(s) declare that they have no competing interests.

## Authors' contributions

FEM developed the hypothesis and wrote most of the manuscript. DRF critically revised the article and wrote guidelines for treatment of CVS patients.

## Pre-publication history

The pre-publication history for this paper can be accessed here:


